# Bacterial Motility Measured by a Miniature Chamber for High-Pressure Microscopy

**DOI:** 10.3390/ijms13079225

**Published:** 2012-07-24

**Authors:** Masayoshi Nishiyama, Seiji Kojima

**Affiliations:** 1 The Hakubi Center, Kyoto University, Yoshida-Ushinomiya-cho, Sakyo-ku, Kyoto 606-8302, Japan; 2 Institute for Integrated Cell-Material Sciences (WPI-iCeMS), Kyoto University, Yoshida-Honmachi Sakyo-ku, Kyoto 606-8501, Japan; 3 Division of Biological Science, Graduate School of Science, Nagoya University, Nagoya 464-8602, Japan; E-Mail: z47616a@cc.nagoya-u.ac.jp

**Keywords:** high-pressure microscopy, bacterial motility, flagellar motor

## Abstract

Hydrostatic pressure is one of the physical stimuli that characterize the environment of living matter. Many microorganisms thrive under high pressure and may even physically or geochemically require this extreme environmental condition. In contrast, application of pressure is detrimental to most life on Earth; especially to living organisms under ambient pressure conditions. To study the mechanism of how living things adapt to high-pressure conditions, it is necessary to monitor directly the organism of interest under various pressure conditions. Here, we report a miniature chamber for high-pressure microscopy. The chamber was equipped with a built-in separator, in which water pressure was properly transduced to that of the sample solution. The apparatus developed could apply pressure up to 150 MPa, and enabled us to acquire bright-field and epifluorescence images at various pressures and temperatures. We demonstrated that the application of pressure acted directly and reversibly on the swimming motility of *Escherichia coli* cells. The present technique should be applicable to a wide range of dynamic biological processes that depend on applied pressures.

## 1. Introduction

Hydrostatic pressure is one of the physical stimuli that characterize the environment of living matter. The deep sea is an environment with particularly high hydrostatic pressure, and life may have originated there about four billion years ago. Hydrostatic pressure is considered to be one of the important stimuli for early forms of life [1,2]. Even at present, many microorganisms thrive in, and may even require, physically or geochemically, high hydrostatic pressure conditions [3,4]. However, application of high pressures is detrimental to most forms of life on Earth, and especially to organisms under ambient pressure conditions. In general, applied pressures change the structure and function of biomolecules *in vivo*, so that it can induce significant changes in the morphology and activity of living cells, such as microorganisms [5,6], sea urchin eggs [7], tissue cells [8], and muscle fibers [9,10].

*Escherichia coli* is a representative research target for studying the mechanism of how hydrostatic pressure affects the activities of biological systems. In previous studies, the major interest in *E. coli* has been on the effects of pressure on cell growth and morphological deformation. At <25 MPa, *E. coli* cells continue to grow and divide [11]. At 30–50 MPa, cell division is inhibited, leading to filamentous elongation of the cell body [12–14]. Cell growth is abolished at >60 MPa [11–13], and cell death results at >150 MPa [15–17]. These results suggest that application of pressure inhibits the activities of living in *E. coli* cells. To study the mechanism of how living organisms adapt to high-pressure conditions, it is necessary to monitor directly the organism of interest under various pressure conditions. Previously, a lot of high-pressure chambers have been developed for not only conventional spectroscopy [18–24], but also optical microscopy [14,25–29].

Here, we report a miniature chamber for high-pressure microscopy. The apparatus developed has two major advantages for microscopic observations. The chamber was equipped with a built-in separator, in which water pressure was properly transduced to that of the sample solution. This mechanism enabled us to drastically reduce the dead volume of buffer solution in the pressure line. Next, the chamber could be settled to a slide glass holder on a commercially available microscope, and allowed us to acquire bright-field and epifluorescence images at various pressures and temperatures. Using this system, we characterized the pressure dependence of the motility of swimming *E. coli* cells and single flagellar motors.

## 2. Results and Discussion

### 2.1. Pressure Dependence of Motility of Swimming *E. coli*

*E. coli* cells sense their environment and swim towards favorable conditions using rotating flagella, a phenomenon known as chemotaxis [30,31], aerotaxis [32], phototaxis [33] or thermotaxis [34–40] depending on the nature of the stimulus. Each flagellum consists of a long (~10 μm), thin (~20 nm), helical filament [41,42] and turns like a screw using a rotary motor at its base [43–47]. The motor can rotate its flagellum in either counterclockwise (CCW, viewed from filament to motor) or clockwise (CW) direction [48]. CCW rotation allows several filaments on a cell to join in a bundle and propel the cell smoothly in solution (a “run”). In contrast, CW rotation forces a filament out of a bundle and leads to a change in swimming direction (a “tumble”) [43]. The switching between CCW and CW rotation enables bacteria to navigate to more favorable environments. Flagellar motility is thought to be one of the most pressure-sensitive cellular processes [49,50]. Recently, we have reported that the swimming fraction and speed of *E. coli* cells decrease with increased pressure [29]. In the present study, we measured the motility of *E. coli* cells under high-pressure conditions and evaluated the performance of the current system.

**Figure 1:**
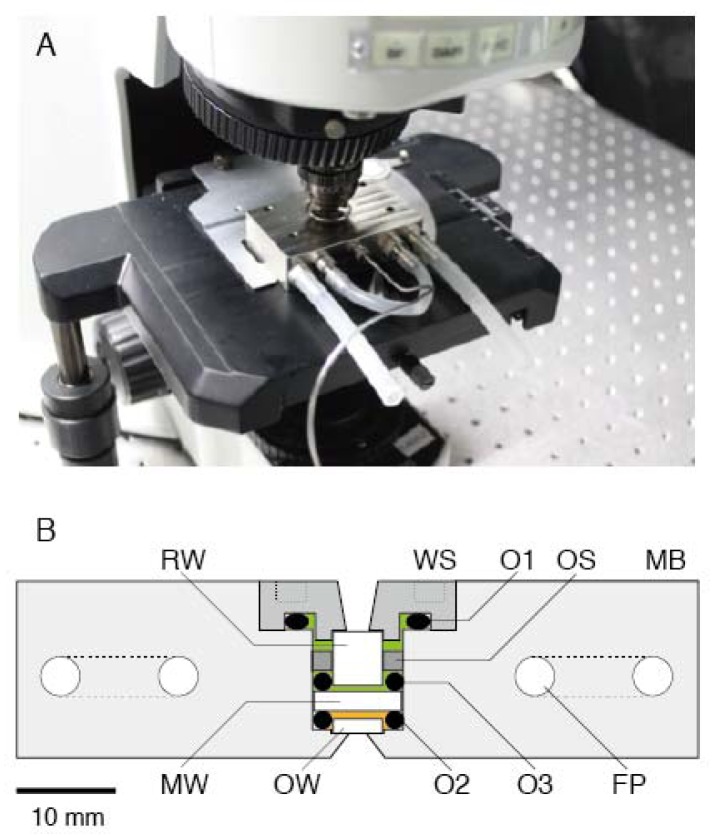
High-pressure microscope. (**A**) Photograph of the high-pressure chamber (HPC) mounted on an upright microscope without any modifications. (**B**) Cross section of HPC. MB, main body; FP; U-shaped flow path; WS, window support; OW, observation window; MW, medium window; RW, rear window; O1, O2 and O3, O-rings. The orange and green areas were filled with assay buffer and distilled water, respectively.

*E. coli* RP4979 cells were diluted in motility medium, and then introduced into the high-pressure chamber (Figure 1A). RP4979 cells lack the switch-inducing CheY protein; therefore, their flagellar motors rotate exclusively in the CCW direction, and cells swim smoothly without tumbling. Under ambient conditions (0.1 MPa and 23 °C), RP4979 cells swam smoothly in solution with a speed of 22 ± 6 μm·s^−1^ (mean ± SD, *n* = 31). The pressure of the chamber was increased up to 80 MPa, and then decreased to 0.1 MPa. At 40 MPa, most cells still swam smoothly, but the average speed decreased to 60% of the initial value at 0.1 MPa. At 60 MPa, many cells still swam, but the swimming speed drastically decreased, and the others just jiggled without showing any translational motion. At 80 MPa, most cells stopped directional swimming and diffused freely in translational and rotational directions. A limited number of cells seemed to show a rolling motion of their cell bodies. The trajectories of the cells at each pressure are similar to our previous results [29].

**Figure 2:**
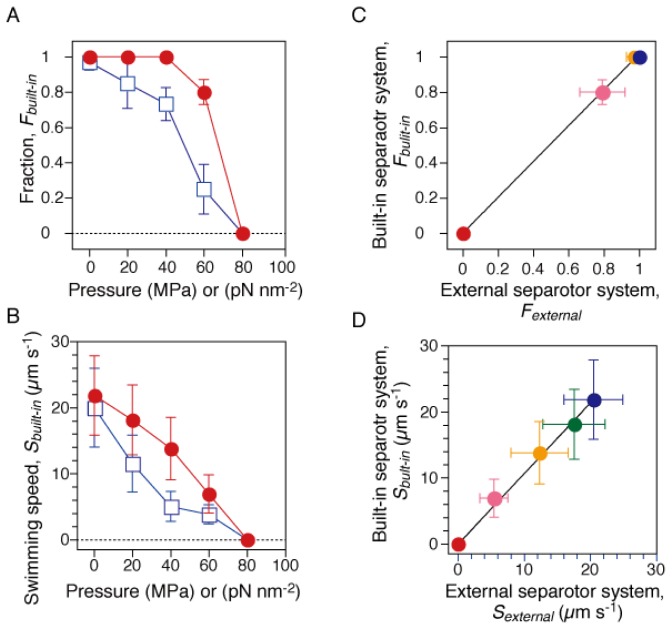
Motility of smooth-swimming cells. The motility assay was performed by two different systems. The current high-pressure chamber was equipped with a “built-in” separator, in which water pressure was properly transduced to that of the sample solution (*See Section 3.1*). On the other hand, the previous one was equipped with an “external” separator [29]. (**A** and **B**) Swimming fraction and speed during the pressurization (closed circles) and depressurization processes (open squares). Swimming fractions, *F_built-in_*, were based on the number of cells that swam with a speed of > 2 μm s^−1^ at each pressure. The speed, *S_built-in_*, was the average value of the swimming cells in **A**. Error bars are the SD. (**C** and **D**) Correlations between the results measured by “built-in” and “external” separator systems. The swimming fraction (**C**) and speed (**D**) at 0.1 (blue), 20 (green), 40 (yellow), 60 (pink) and 80 MPa (red). The plots in **C** and **D** were fitted to lines with slopes of 1.02 ± 0.01 and 1.07 ± 0.02 (± fitting error), respectively.

The fraction and speed of the swimming cells during the pressurization and depressurization processes were analyzed. We selected the cells that swam with a speed of >2 μm·s^−1^ in the focal plane, and calculated their fraction among all cells, and their average speed. Figure 2A,B show that these two parameters decreased with increased pressure and reached zero at 80 MPa. Both the swimming fraction and speed showed significant hysteresis between the pressurization and depressurization processes, although the cells eventually recovered their initial motility sometime after the pressure was released.

Next, we repeated the motility assay of RP4979 cells derived from the same cultures by using a previous high-pressure microscope [29]. The previous system was equipped with a large external separator [cylindrical tube; *Φ* = 60 mm, *L* = 94 mm, stainless steel (SUS630)] [27], in which the pump pressure was transduced to that of the buffer solution. Similar results were obtained as with the previous system (data not shown). Figure 2C,D summarize the correlations between the results measured by the current and previous systems. The plots of the swimming fraction and speed were fitted to lines with slopes of 1.02 ± 0.01 and 1.07 ± 0.02 (mean ± fitting error), respectively. Thus, the results clearly demonstrated that the current system properly transduced the pump pressure to that of the buffer solution, and then inhibited the motility of swimming cells.

In summary, we performed a motility assay of swimming *E. coli* cells at high pressure, and evaluated the performance of the developed system. We analyzed the swimming fraction and speed of *E. coli* cells, but these parameters largely depended on the experimental conditions, such as the strain, cultivation conditions, or solution viscosity [29,49,50]. To examine the performance of the system in more detail, we will develop a novel probe responding sensitively to the applied pressure.

### 2.2. Torque Generation of Single Flagellar Motors in Tethered Cells

The bacterial flagellar motor is a reversible molecular motor that converts a specific ion flux to the rotation of a flagellum [43–47]. The rotational motion is composed of regular 26 steps per single turn [51,52]. The coupling ion differs according to the type of motor and/or bacterial species. *E. coli* and *Salmonella enterica* motors use H^+^ [53], and those of the alkalophilic *Bacillus* and marine *Vibrio* species utilize Na^+^ [54,55]. The flagellar motor consists of a rotor and multiple stator units. The rotor spins relative to the cell body and its rotation transduces to the flagellum, whereas the stator units are anchored to the cell wall. The rotational speed is affected by physical and chemical conditions, such as viscous load [55–58], temperature [59–62], pH [63,64] and solvation [61]. The application of pressure is also expected to modulate the torque generation processes. Here, we studied the motility of bacterial flagellar motors under high-pressure conditions.

**Figure 3:**
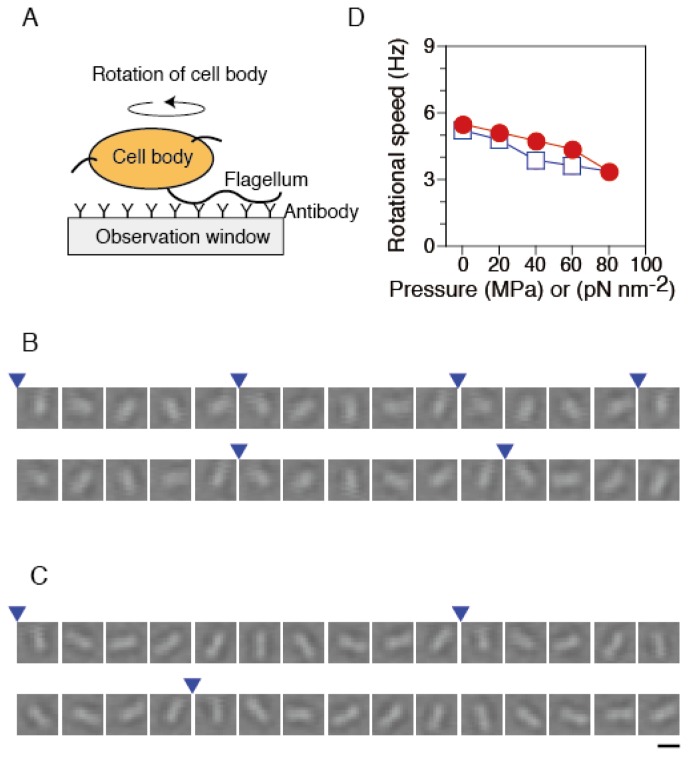
Rotational speed of single flagellar motors. (**A**) Schematic drawing of the experimental system (not to scale). (**B** and **C**) Sequential bright-field images of the same rotating tethered cell at 33 ms intervals at 0.1 MPa (**B**) and 80 MPa (**C**). The images are displayed after processing contrast enhancement and brightness offset. Blue arrowheads indicate completion of a turn. Scale bar, 2 μm. (**D**) The plots are mean values (*n* = 52) of the rotational speed in the pressurization (circles) and depressurization processes (diamonds). Each speed was obtained from the rotation number during 10 s. Data for the motors that were in the stop state were excluded from calculations of the speed.

To monitor the rotation of single flagellar motors, we performed a rotating tethered-cell assay [48], using strain RP4979. A single flagellar filament protruding from each cell was attached to the surface of the observation window, OW, in the chamber via antibody of its flagellum (Figure 3A). We tracked the rotation of the same cells under various pressure conditions. Under ambient conditions (0.1 MPa and 23 °C), motors rotated smoothly (Figure 3B). Even at 80 MPa, most cells remained anchored to the OW, and then the motor still rotated in a CCW direction (Figure 3C).

Systematic analysis was performed to characterize the pressure dependence of the rotational speed of the motor in tethered cells. We selected the motors that rotated smoothly after the release of pressures again. The rotational speed of the same motors was tracked, when the pressure was increased and then decreased in a stepwise manner. Figure 3D summarizes the pressure-speed relation of single flagellar motors in the pressurization and depressurization processes. At ambient pressure (0.1 MPa), the motor rotated with a speed of 5.5 ± 0.5 s^−1^ (mean ± SE, *n* = 52). The rotational speed decreased with increased pressure, but even at 80 MPa, about 90% of motors still rotated at 3.4 ± 0.5 Hz (mean ± SE, *n* = 47). The pressure-speed curve showed an upper concave relation. After the pressure was released, the motors eventually recovered the initial motility. The pressure-speed relation did not show significant hysteresis between pressurization and depressurization processes. These experimental results are consistent with our previous studies, in which we analyzed the pressure dependence of the rotational speed of the cells that express FliC-sticky filaments [46].

Our results showed that the motility of swimming *E. coli* cells was very sensitive to hydrostatic pressure (Figure 2); however, the motor function was relatively robust to pressure (Figure 3). What causes these differences? The robustness of the motor function against pressure is thought to be because torque is generated by the limited numbers of protein units in the motor via relatively simple mechanisms, although a flagellar motor is composed of a large number of protein molecules [43–47]. The flagellar motor consists of a rotor and multiple stator units. Four copies of MotA and two of MotB form a proton channel complex and function as a component of a stator unit [65–68]. FliG forms a series of rings in the rotor [69–71] and interacts with the MotA/B complex to generate torque for rotation [72,73]. Analyses of site-directed mutagenesis have shown that the replacement of charged residues in MotA/B or FliG proteins causes defective torque generation, suggesting that the electrostatic interaction is crucial for proton translation and intermolecular interaction between MotA/B and FliG [73–75]. Our previous studies have suggested that the applied pressure decreases the rate of proton translocation in the mechanochemical energy conversion, but does not dissociate MotA/B from FliG [46]. The detailed mechanism could be elucidated by measuring the torque–speed relation of a single flagellar motor at high pressure [55–57,76,77].

In summary, we demonstrated a motility assay of single flagellar motors at high pressure, and characterized the pressure dependence of the motor rotation in *E. coli* cells. The present technique could be combined with other advanced microscope techniques [78–85]. High-pressure microscopy will be extended to study not only the flagellar rotation, but also other cellular processes [2,86,87].

## 3. Experimental Section

### 3.1. High-Pressure Chamber and Microscope

Figure 1B shows a cross section of a high-pressure chamber (70 × 43 × 18 mm; Sasahara Giken, Kyoto, Japan). The main body (MB) and window support (WS) were made of nickel alloy (Hastelloy C276). The MB was equipped with two U-shaped flow paths (FPs) for running the temperature-regulated water of the thermostat bath. The WS was screwed into the MB, and the inside of the chamber was sealed by an O-ring (O1). The inside of the chamber was separated by a medium window (MW; *Φ* = 8.9 mm, *t* = 2.0 mm, quartz; Sasahara Kogaku, Kyoto, Japan). The MW was supported by two O-rings (O2 and O3) and its support (OS). The gap between the observation window (OW) and MW was filled with buffer solution (orange area in Figure 1B) and its volume was 0.1 mL, which was 1/100 of the previous pressure apparatus [27]. The remaining cavity was filled with distilled water (green area in Figure 1B) and connected to a high-pressure pump (HP-150; Syn Corporation, Kyoto, Japan) via a long spring-like 1/16-inch stainless tube. The water pressure was transduced to that of the buffer solution through depression of the MW. The water pressure in the pressure line was measured with a high-pressure gauge (PG-2TH; Kyowa, Kyoto, Japan). Our apparatus could be used for applications of pressure up to 150 MPa, which was constrained by performance of the hand pump. The withstanding pressure was 1.5-fold higher than the water pressure in the deepest part of the Mariana Trench, Challenger Deep (10,900 m in depth). This level of ability to withstand pressure is sufficient for studying almost all biological activities on Earth.

The chamber was equipped with two more optical windows, that is, OW (*Φ* = 5 mm, *t* = 1.5 mm, BK7; Sasahara Kogaku) and rear window (RW; *Φ* = 5 mm, *t* = 5.5 mm, BK7; Sasahara Kogaku). Two windows (OW and RW) were attached to the MB and WS, respectively, by epoxy resin. The OW was made of BK7 because this material was found to be suitable for microscopic observation and for preparing appropriate surface conditions for our experiments (the numerical index of sapphire or quartz is far from that of glass, which means that these materials are not suited for acquiring better images). Microscopic observations in the chamber were carried out through an OW. The aperture diameter and critical angle were 1.5 mm and 76°, respectively. The numerical aperture (NA) at the objective lens side was 0.6.

The miniature chamber was combined with a commercially available upright microscope (BX51; Olympus, Tokyo, Japan) on a vibration-free table (AS-II 1510TM; Nippon Boushin Industry Co., Shizuoka, Japan). The chamber was settled on the microscope stage with a conventional slide holder (Figure 1A). Microscopic observation was done by a long-working distance objective lens (OL; NA 0.6, WD 3 mm, LUCPLFLN40×; Olympus). Bright-field and epifluorescence images were acquired without any modifications (data not shown).

### 3.2. Bacterial Strains

Here, we used *E. coli* strains RP437 [88] and RP4979 [89]. Strain RP437 was a wild-type strain for motility and chemotaxis. Strain RP4979 (*ΔcheY*) was derived from strain RP437. To focus on the pressure dependence of the motor function in swimming *E. coli* cells, strain RP4979, rather than strain RP437, was used for the motility assays. The flagellar motor in RP4979 cells rotates exclusively in the CCW direction, and then cells swim smoothly without tumbling.

### 3.3. Purification of *E. coli* Flagellin and Preparation of the Anti-Flagellin Antibody

**Figure 4:**
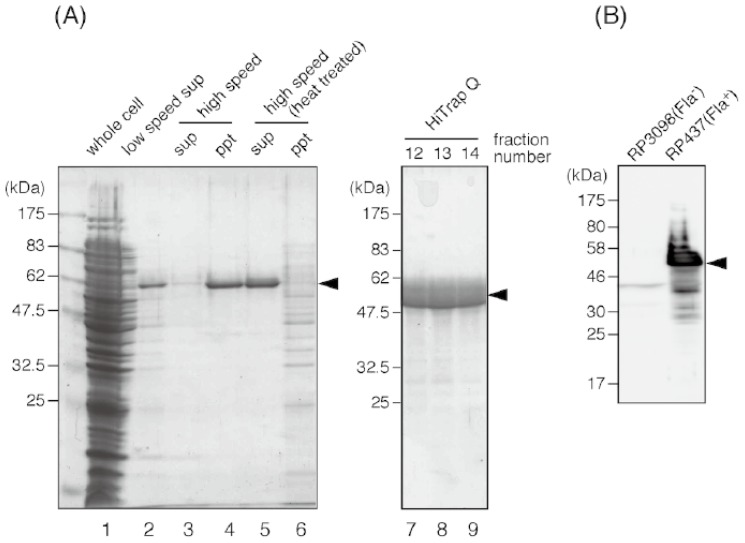
Purification of the *E. coli* flagellin. Protein samples in each purification step are resolved on a Coomassie-stained 12% SDS-PAGE gel. (**A**) Lane 1, whole cell lysate; lane 2, supernatant of low speed centrifugation after shearing flagella by a blender; lane 3 and 4, supernatant and pellet of the ultracentrifugation of the flagella-containing suspension; lane 5 and 6, supernatant and pellet of the ultracentrifugation after heat treatment; lane 7 to 9, peak fractions of the HiTrap Q column. (**B**) Immunoblot detection of flagellin by using the antibody raised against the purified *E. coli* flagellin. A strong flagellin band can be seen for the whole cell sample of the wild-type *E. coli* strain RP437, but not for that of the strain RP3098, which does not produce any flagellar proteins. An arrowhead indicates *E. coli* flagellin (51 kDa).

To obtain the anti-flagellin antibody, we purified the *E. coli* flagellin from strain RP437. The cells were grown on LB plates overnight at 37 °C. Cells were scraped from the plates and suspended in 20 mM Tris-HCl (pH 8.0) at the concentration of 10 mL/g wet pellet. The cell suspension was intensively blended for 3 min by a homogenizer (Polytron PT3000, 8000 revolution per minute) to shear flagella, and then centrifuged at 5000*g* for 10 min to spin down the cells. After this step, flagellin became a major protein in this supernatant (Figure 4A, lane 2).

The supernatant was ultracentrifuged at 101,500*g* for 1 h to precipitate flagella (Figure 4A, lane 4). The pellet was suspended in 20 mM Tris-HCl (pH 8.0) and heated. When the temperature reached 75 °C, the suspension was further incubated for 15 min, then immediately cooled down by ice-cold water. After this heat treatment, the depolymerized, soluble flagellin was separated from insoluble materials by ultracentrifugation (101,500*g* for 1 h, Figure 4A lane 5). Flagellin was further purified by using an anion-exchange column (HiTrap Q, GE Healthcare Japan, Tokyo, Japan) and eluted with a linear 0 to 300 mM gradient of NaCl. Peak fractions (Figure 4, lane 7 to 9) were collected and concentrated to 11 mg/mL by ultrafiltration using an Amicon Ultra device (Millipore, Billerica, MA, USA). At this final stage, flagellin was more than 90% pure and appeared as a main single band on the SDS-PAGE gel. The band corresponding to flagellin was excised and the protein was extracted from the gel by crushing it, then mixed with adjuvant and injected into rabbits for immunization.

The rabbit anti-flagellin antibody was produced by Keary Co. (Osaka, Japan). We found aggregations of RP437 cells by their flagella in the presence of 1:1000 dilution of anti-flagellin antibody. Moreover, the antibody can be used for immunoblot, to specifically detect the *E. coli* flagellin (Figure 4B). For immunoblot, the whole cell samples of the non-flagellated strain RP3098 and wild-type strain RP437 were prepared from the cell culture grown in Tryptone broth (1% Bacto tryptone, 0.5% NaCl), and an immunoblot was carried out as described previously [90]. The prepared anti-flagellin antibody is available to interested parties upon request.

### 3.4. Motility Assays

Motility of strain RP4979 was measured in this study. Cells were cultured from frozen stocks to the late logarithmic phase at 30 °C in Tryptone broth as described in Section 3.3. The grown cells were adequately diluted with the motility medium (10 mM Tris, pH 7, 0.1 mM EDTA) and enclosed in the current or previous high-pressure chamber. We monitored the cells that swam near the OW in the chamber.

In a tethered-cell assay [91], the flagella of RP4979 cells were sheared as described previously [29]. A single flagellum protruding from a cell was attached to the surface of the OW via antibody of its flagellum (Figure 3A). We tracked the motor rotation of the same single cells under various pressure conditions.

The bright-field image of the swimming or rotating tethered cells near the OW was acquired by a CCD camera at 30 frames s^−1^ and stored in a computer. The hydrostatic pressure within the chamber was increased to 80 MPa in increments of 20 MPa and decreased by similar steps. The increment of 20 MPa of pressure was performed within a few seconds. We readjusted the position of the chamber in *x*- and *y*-directions, and the objective lens in *z*-direction, and then acquired the microscopic images during about 2 min. Pressure within the chamber could be decreased by opening a pressure-regulating valve. The total elapsed time for pressure treatment of a population of cells was about 30 min. The pressure was regulated with an accuracy of ± 1 MPa. The experimental temperature was kept at 23 ± 1 °C. After release of the pressure, all cells were removed from the chamber and the assay was repeated using fresh cells. We used two different chambers for high-pressure microscopy, but the motility assays were performed by the same procedures. All motility assays were performed within 2 h, and repeated by using more than two different cultures. All images were analyzed offline, using commercial tracking software (G-track; G-Angstrom, Sendai, Japan).

## 4. Conclusions

Here, we report a miniature chamber for high-pressure microscopy. The chamber was equipped with a built-in separator, in which water pressure was properly transduced to that of the sample solution. This mechanism enabled us to drastically reduce the dead volume of buffer solution in the pressure line. The apparatus developed here can be used with a commercially available microscope, and enables us to conveniently acquire microscopic images at high-pressure. Application of pressure is a powerful method for modulating intermolecular interactions between protein and water molecules. The present technique could be extended to study the mechanisms by which isolated molecular machines are affected by the application of high pressure.
